# Inhibition of Neuronal Necroptosis Mediated by RIPK1 Provides Neuroprotective Effects on Hypoxia and Ischemia In Vitro and In Vivo

**DOI:** 10.3390/ijms23020735

**Published:** 2022-01-10

**Authors:** Elena V. Mitroshina, Maria M. Loginova, Roman S. Yarkov, Mark D. Urazov, Maria O. Novozhilova, Mikhail I. Krivonosov, Mikhail V. Ivanchenko, Maria V. Vedunova

**Affiliations:** 1 Institute of Biology and Biomedicine, Lobachevsky State University of Nizhni Novgorod, 23 Prospekt Gagarina, 603950 Nizhny Novgorod, Russia; pandaagron@yandex.ru (M.M.L.); roman.sultanov.96@mail.ru (R.S.Y.); urazov@neuro.nnov.ru (M.D.U.); masananov@yandex.ru (M.O.N.); mvedunova@yandex.ru (M.V.V.); 2 Institute of Information Technology, Mathematics and Mechanics, Lobachevsky State University of Nizhni Novgorod, 23 Prospekt Gagarina, 603950 Nizhny Novgorod, Russia; mike_live@mail.ru (M.I.K.); ivanchenko.mv@gmail.com (M.V.I.)

**Keywords:** RIPK1, necrostatin-1, primary hippocampal cultures, functional neural network activity, neuroprotection, ischemia, hypoxia

## Abstract

Ischemic brain injury is a widespread pathological condition, the main components of which are a deficiency of oxygen and energy substrates. In recent years, a number of new forms of cell death, including necroptosis, have been described. In necroptosis, a cascade of interactions between the kinases RIPK1 and RIPK3 and the MLKL protein leads to the formation of a specialized death complex called the necrosome, which triggers MLKL-mediated destruction of the cell membrane and necroptotic cell death. Necroptosis probably plays an important role in the development of ischemia/reperfusion injury and can be considered as a potential target for finding methods to correct the disruption of neural networks in ischemic damage. In the present study, we demonstrated that blockade of RIPK1 kinase by Necrostatin-1 preserved the viability of cells in primary hippocampal cultures in an in vitro model of glucose deprivation. The effect of RIPK1 blockade on the bioelectrical and metabolic calcium activity of neuron-glial networks in vitro using calcium imaging and multi-electrode arrays was assessed for the first time. RIPK1 blockade was shown to partially preserve both calcium and bioelectric activity of neuron-glial networks under ischemic factors. However, it should be noted that RIPK1 blockade does not preserve the network parameters of the collective calcium dynamics of neuron-glial networks, despite the maintenance of network bioelectrical activity (the number of bursts and the number of spikes in the bursts). To confirm the data obtained in vitro, we studied the effect of RIPK1 blockade on the resistance of small laboratory animals to in vivo modeling of hypoxia and cerebral ischemia. The use of Necrostatin-1 increases the survival rate of C57BL mice in modeling both acute hypobaric hypoxia and ischemic brain damage.

## 1. Introduction

With ischemic damage, pathological cascades, leading to disturbances in the functional and metabolic activity of the brain’s neural networks, develop. It eventually leads to the launch of necrotic and apoptotic processes and the death of structural and functional elements of the neural network [1,2,3]. The key damaging factors of ischemia are hypoxia and energy substrate deficiency (glucose deprivation). Ischemic stroke is one of the leading causes of death and disability of population. Important to note, that hypoxic and ischemic damage are not only isolated pathologies and often accompany various neurodegenerative diseases, traumatic brain injury, etc. Perinatal hypoxic and ischemic injuries are also widespread and among the leading causes of infant mortality and invalidation. In recent years, kinase enzymes, which regulate a variety of cellular functions by phosphorylating target proteins, have been of great interest in the search for molecular targets to create possible therapeutic agents for the correction of ischemic damage. Kinases affect energy metabolism (glycogen and glucose utilization) in the cell, participate in the synthesis of coenzymes and nucleotide metabolism, and are also the most important components of signaling cascades responsible for maintaining cell viability [4,5,6].

Receptor-interacting protein kinase 1 (RIPK1) belongs to the class of serine/threonine kinases and plays an important role in the signaling pathways triggered by death receptors. Activated death receptors (DR) cause RIPK1 phosphorylation, which leads to the formation of the RIPK1-RIPK3 complex called complex IIb. The formation of this complex leads to phosphorylation of the mixed lineage kinase domain-like protein (MLKL), which ultimately leads to MLKL-mediated necroptosis resulting in plasma membrane destruction and cell lysis [7]. In the case of a mitochondria dysfunction, the production of reactive oxygen species increases significantly. Reactive oxygen species contribute to the activation of the inflammatory cascade, disruption of the blood–brain barrier permeability, and vascular endothelial dysfunction, ultimately leading to neuronal dysfunction and neurodegeneration [8]. Of great interest is the information obtained on retinal cells, showing that oxidative stress can lead to aberrant expression of genes involved in the regulation of ferroptosis and can trigger this type of cell death [9,10]. A number of recent studies have described the protective effects of this kinase blockade with the selective necroptosis inhibitor necrostatin-1 (Nec-1). For example, it has been shown that RIPK inhibition is able to attenuate ischemic brain injury, as well as the neurological manifestations of ischemic stroke with occlusion of the middle cerebral artery in rats [11]. A similar effect has been shown for genetic knockdown of both RIPK1 and RIPK3, the second key regulator of necroptosis, and the downstream effector MLKL, in modeling traumatic brain injury [12].

However, there are still very few data on the neuroprotective effects of RIPK1 inhibition in ischemic brain injury. To date, there are no studies that have examined the effect of RIPK1 kinase blockade on the functioning of neural networks in the brain. Key brain functions depends not on individual neurons but rather on their functional ensembles. A neural network is the minimal functional unit of the nervous system, and it ensures the implementation of higher cognitive functions, including memory, consciousness, etc. So, it is essential to investigate the functional network activity of neural cells. Primary dissociated cell cultures of different brain areas are one of the most adequate and modern experimental models for the studying of neural networks’ functional activity. The cultures allow to study cell and network reactions on both stress conditions and neuroprotector implication in chronic experiment. Moreover, primary dissociated neural cultures obtained from mouse embryos on late gestational period pass the main ontogenetic stages of neural-network formation in the process of cultivation [13,14,15].

Our work aimed at studying the effects of receptor-interacting protein kinase 1 (RIPK1) inhibition on the functional activity of neural networks in primary hippocampal cultures in modeling key factors of ischemia in vitro. Moreover, we investigated the neuroprotective effect of Nec-1 in vivo when modeling acute hypobaric hypoxia and ischemic brain injury.

## 2. Results

### 2.1. Studies of the Effect of RIP1 Kinase Blockade on the Functional Activity of Neural Networks in Modeling Ischemic Factors In Vitro

**Figure 1:**
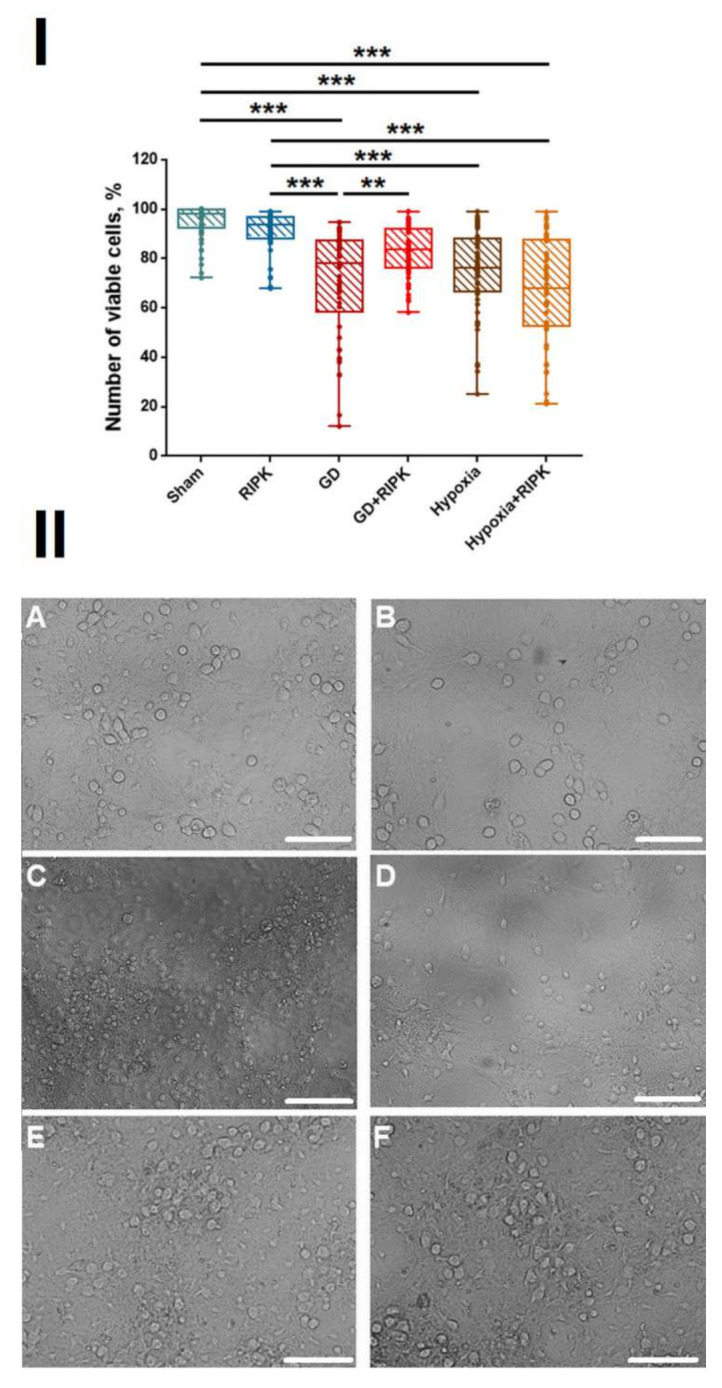
Cell viability of primary hippocampal cultures on day 7 posthypoxia or post-GD modeling. (**I**)—analysis of cell viability. ** *p* ≤ 0.01, *** *p* ≤ 0.001. Two-way ANOVA followed by Bonferroni post-hoc test for multiple comparisons. F= 27.79. Sham vs. RIPK—NS; Sham vs. GD *p* ≤ 0.001; Sham vs. GD + RIPK *p* = 0.004; GD vs. GD + RIPK *p* = 0.008; Sham vs. Hypoxia *p* ≤ 0.001. Sham vs. Hypoxia + RIPK *p* ≤ 0.001; Hypoxia vs. Hypoxia + RIPK NS. (**II**)—Representative examples of morphological changes in primary hippocampal cell cultures on day 7 after modeling ischemic factors. (**A**)—Sham, (**B**)—RIPK1, (**C**)—Glucose Deprivation, (**D**)—GD + RIPK1, (**E**)—Hypoxia, (**F**)—Hypoxia + RIPK1. Scale bar 50 mcm.

The first stage of the work was to study the effect of RIPK1 inhibition by necrostatin-1 under physiological conditions. Inhibition of RIPK1 kinase under normal conditions did not lead to significant changes in the morphology and viability of cells in primary hippocampal cultures (Figure 1).

One of the most common brain injuries is ischemic stroke. Ischemia is a complex multifactorial disease associated with pathophysiological processes, the main active chains of which are substrate and oxygen starvation. Therefore, we assessed the neuroprotective potential of the Nec-1, RIPK1 inhibitor, using two models: modeling glucose deprivation and acute normobaric hypoxia in vitro as key damaging factors of ischemia. There was a significant decrease in the number of viable cells after modeling both damaging factors to 73.96 ± 2.9% in the “GD” group and 77.94 ± 1.75% in the “Hypoxia” group, respectively. Necroptosis inhibitor Nec-1 was shown to effectively protect cells from damage caused by energy substrate deficiency (“GD + RIPK” 86.82 ± 1.49%) but does not affect the viability in hypoxic damage (“Hypoxia + RIPK” 66.09 ± 4.61%).

The main objective of our study was to assess the functional activity of the neural-glial network after exposure to damaging ischemic factors. The registration of calcium concentration dynamics in the cytoplasm of nerve cells is considered one of the most informative approaches for this purpose [16]. Calcium ions play a key role in the signal transmission between nerve cells. Analysis of calcium events in nerve cells allows to reconstruct neuronal activity and map neuron-glial networks with cellular and subcellular resolution. Assessment of the effect of RIP1 kinase blockade on functional neural-network activity in the postischemic period is extremely important since it is a unique provider of a comprehensive picture of the preservation of the functional architecture of the neuron-glial network.

**Figure 2:**
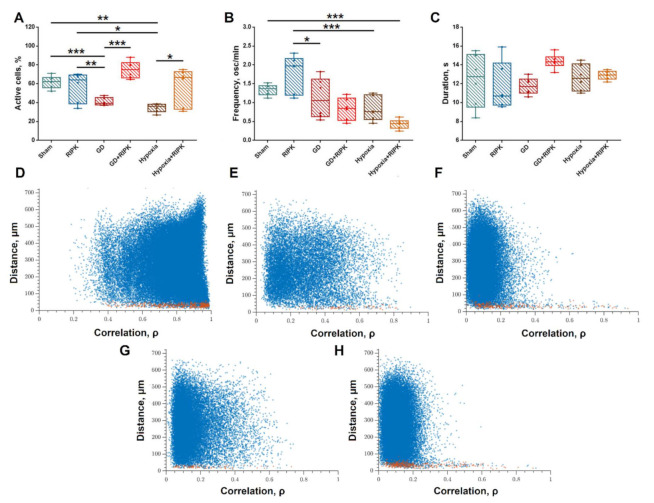
Analysis of calcium activity. (**A**–**C**)—Main parameters of calcium activity of primary hippocampal cultures together with the blockade of intracellular kinases. (**A**)—Active cells—the cell number with at least one recorded oscillation divided by the total cell number; F= 13.68. Sham vs. RIPK—NS; Sham vs. GD *p* ≤ 0.001; Sham vs. GD + RIPK—NS; GD vs. GD + RIPK *p* < 0.001; Sham vs. Hypoxia *p* = 0.005. Sham vs. Hypoxia + RIPK—NS; Hypoxia vs. Hypoxia + RIPK *p* = 0.026; (**B**)—Frequency. osc/min. F= 10.91. Sham vs. RIPK—NS; Sham vs. GD NS; Sham vs. GD + RIPK—NS; GD vs. GD + RIPK NS; Sham vs. Hypoxia NS. Sham vs. Hypoxia + RIPK— *p* = 0.001; Hypoxia vs. Hypoxia + RIPK NS; (**C**)—Duration of calcium oscillation. F= 1.93. NS. * *p* ≤ 0.05, ** *p* ≤ 0.01, *** *p* ≤ 0.001 Two-way ANOVA followed by Bonferroni post-hoc test for multiple comparisons. (**D**–**H**)—Relationship between the level of signal similarity and the distance between pairs of cells. (red dots are pairs of neighboring cells; blue dots are pairs of distant cells). The *x*-axis is a measure of the similarity between pairs of cells, calculated as the maximum value of cross-correlation, taking into account the signal delays. Each point is determined by a coordinate on the *x*-axis—the level of signal similarity—and on the *y*-axis—the distance between a pair of corresponding cells. (**D**)—Sham, (**E**)—GD, (**F**)—GD + RIPK1, (**G**)—Hypoxia, (**H**)—Hypoxia + RIPK1. The *x*-axis is the measure of similarity between pairs of cells, calculated as the maximum value of cross-correlation, taking into account signal delays. Each point is determined by a coordinate on the *x*-axis—the level of signal similarity—and on the *y*-axis—the distance between a pair of corresponding cells.

The study of functional calcium activity was performed on day 7 after modeling ischemic factors to assess the long-term consequences of ischemic injury. It was shown that at 21 DIV 59.28 ± 6.05% of cells in the hippocampal cell culture were active, the duration of calcium oscillations was 12.37 ± 1.16 s, and the frequency was 1.34 ± 0.14 osc/min (Figure 2A–C). Exposure to both ischemic factors leads to the inhibition of calcium activity and a significant decrease in the number of cells in which Ca^2+^ events are registered (GD—41.62 ± 1.71,%; Hypoxia—35.91 ± 1.05%) and is consistent with previously published data [17]. The duration and frequency of calcium oscillations in modeling glucose deprivation and hypoxia did not change significantly.

The application of the RIPK kinase inhibitor necrostatin-1 under normal conditions had no effect on the main parameters of calcium activity. When both glucose deprivation and hypoxia were modeled, the use of inhibitor Nec-1 led to an increase in the number of cells in which calcium oscillations were recorded, compared to the corresponding ischemic factor (Figure 2). Moreover, in the “GD + RIPK1” group, the proportion of cells exhibiting activity was significantly higher than in intact cultures (74.81 ± 5.15). In the “Hypoxia + RIPK1” group, the number of active cells remained at the level of intact cultures.

It is interesting to note that the use of Nec-1 in the modeling of both damaging ischemic factors led to a significant increase in the duration of calcium oscillations in comparison with the "GD" and "Hypoxia" groups, respectively. There was also a decrease in the frequency of calcium events during RIPK1 blockade when modeling ischemic factors.

Thus, the use of Nec-1 in the modeling of hypoxic injury allows to maintain spontaneous calcium activity at the level of intact cultures, in the modeling of acute hypoxia in vitro—by the number of active cells. The RIPK1 blockade in the modeling of glucose deprivation led to an increase in the number of cells exhibiting activity compared to the intact group; however, the frequency of calcium oscillations significantly decreased.

**Figure 3:**
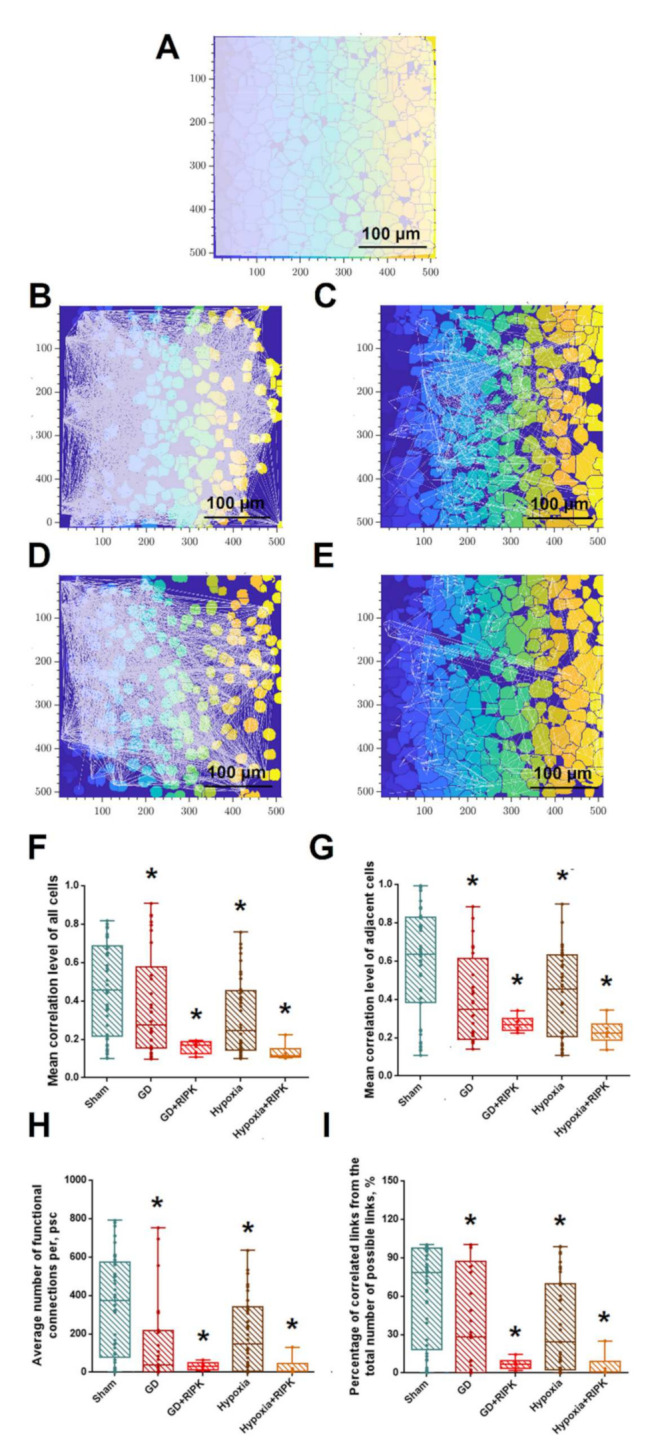
(**A**–**E**) Representative correlation network graphs with a threshold of >0.3. (**A**)—Sham, (**B**)—GD, (**C**)—GD + RIPK1, (**D**)—Hypoxia, and (**E**)—Hypoxia + RIPK1. (**F**–**I**)—Reorganization of neuron-glial network activity in primary hippocampal cultures: (**F**)—the level of correlation of all cells. F= 5.42. Sham vs. GD *p* = 0.044; Sham vs. GD + RIPK *p* = 0.009; GD vs. GD + RIPK—NS; Sham vs. Hypoxia *p* = 0.038. Sham vs. Hypoxia + RIPK *p* = 0.024; Hypoxia vs. Hypoxia + RIPK—NS; (**G**)—the level of correlation of neighboring cells. F= 6.19. Sham vs. GD *p* = 0.024; Sham vs. GD + RIPK *p* = 0.007; GD vs. GD + RIPK—NS; Sham vs. Hypoxia *p* = 0.016. Sham vs. Hypoxia + RIPK *p* = 0.041; Hypoxia vs. Hypoxia + RIPK—NS; (**H**)—the number of functional connections per cell. F= 6.54. Sham vs. GD *p* = 0.029; Sham vs. GD + RIPK *p* = 0.012; GD vs. GD + RIPK—NS; Sham vs. Hypoxia *p* = 0.007. Sham vs. Hypoxia + RIPK *p* = 0.014; Hypoxia vs. Hypoxia + RIPK—NS; (**I**)—the percentage of correlated connections of the maximum possible number of connections. F= 6.35. Sham vs. GD *p* = 0.043; Sham vs. GD + RIPK *p* = 0.005; GD vs. GD + RIPK—NS; Sham vs. Hypoxia *p* = 0.089. Sham vs. Hypoxia + RIPK *p* = 0.007; Hypoxia vs. Hypoxia + RIPK—NS. * *p* ≤ 0.05. One-way ANOVA with repeated-measures and Bonferroni test for multiple comparisons.

The study of the features of the reorganization of neural network activity is of particular interest. The use of the original algorithms developed by us for the analysis of the connectivity parameters of calcium dynamics in cell culture, based on the computer vision methods [18,19], allowed us to characterize the functional architecture of the neuron-glial network. Figure 3A–E shows the dependence between the level of calcium activity correlation and the distance between pairs of cells in culture. Adjacent pairs of cells whose somas are in direct contact with each other are shown with red dots, and distant cells are shown in blue. Based on previous studies, a significant level of correlation, indicating the formation of a functional connection between cells, is the correlation of more than 0.3 [18]. Under normal conditions, both neighboring (0.63 [0.38; 0.82], Figure 2G) and distant (0.46 [0.22; 0.68], Figure 2F) cells have a high level of correlation of calcium event dynamics. Thus, a developed dynamic neuron-glial network characterized by correlated calcium dynamics is formed by primary hippocampal cultures under physiological conditions.

Visualization of functional connections between the elements of the neuron-glial network in the form of a directed graph is shown in Figure 3. Exposure to ischemic factors leads to a significant loss of functional connections between the neural-glial networks (Figure 3A,B,D). Of great interest are our findings that despite the ability of Nec-1 to maintain the number of cells generating spontaneous calcium oscillations associated with stress, the number of functionally significant connections between them decreases significantly, which can be seen in the graphs presented in Figure 3C,D.

After exposure to both damaging factors, the degree of activity correlation between neighboring cells decreases significantly in the late period. The correlation of calcium dynamics between neighboring cells was 0.35 [0.2; 0.56] in the “Glucose deprivation” group and 0.45 [0.22; 0.63] in the “Hypoxia” group (Figure 3G). At the same time, hypoxia has a more pronounced uncoupling effect on the neuron-glial network, which is manifested in a significant decrease in the degree of activity correlation between all pairs of cells in culture (0.27 [0.15; 0.53] in the “Glucose deprivation” group and 024 [0.15; 0.45] in the “Hypoxia” group). It indicates a loss of connections and impaired signaling between nerve cells in the network.

To confirm this assumption, we analyzed the number of functional connections formed by cells in vitro. In intact cultures, it was 372.08 [93.87; 572], which is 78.25 [30.01; 96.96]% of the maximum possible. The maximum possible number of functionally significant connections is considered to be the number in which each cell in the culture would be connected with each other cell (“all to all”). Under the influence of stress factors, the number of functionally significant cellular connections decreases significantly: under GD—39.38 [2.36; 210.24]; under hypoxia—147.47 [8.12; 334.33]. There was a decrease in the share of correlated relationships from the total number of possible relationships (“Sham”—78.25 [30.01; 96.96], “GD”—28.01 [0.57; 82.07], and “Hypoxia”—24.26 [3.39; 69.73]).

Despite the fact that the application of the RIPK1 inhibitor preserved the number of cells exhibiting calcium activity, the network parameters of calcium dynamics significantly decreased and did not differ from those of the “GD” and “Hypoxia” groups. There was a significant decrease in all the considered correlation characteristics in comparison with the “Intact” group.

**Figure 4:**
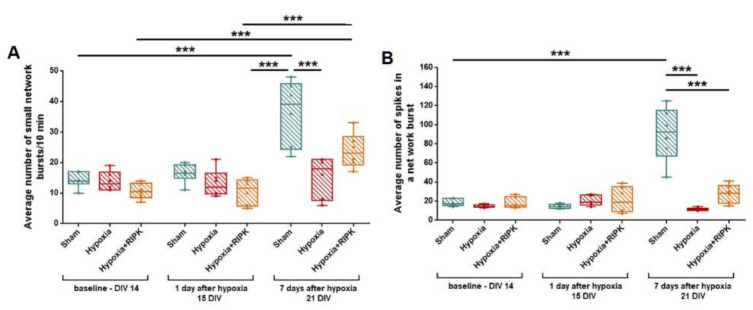
The main parameters of spontaneous bioelectrical activity of primary neuronal cultures associated with kinase blockade and in the modeling hypoxia in vitro. (**A**)—Average number of small network bursts/10 min. DIV 14–NS, DIV 15–NS, DIV 21—F= 10.36. Sham vs. Hypoxia *p* = 0.001. Sham vs. Hypoxia + RIPK—NS. (**B**)—Average number of spikes in a network burst. DIV 14–NS. DIV 15–NS. DIV 21–F = 34.32. Sham vs. Hypoxia *p* < 0.001. Sham vs. Hypoxia + RIPK—*** *p* ≤ 0.001.Two-way ANOVA followed by Bonferroni post-hoc test for multiple comparisons.

The preservation of a significant number of cells exhibiting calcium activity during RIPK1 inhibition in modeling of glucose deprivation and hypoxia, as well as an increased duration of calcium oscillations, can occur if only astrocytes exhibit functional activity in the postischemic period and neuronal activity is eliminated. Since astrocytes are electrically non-excitable cells, the functional activity and connectivity of neurons can be assessed by recording spontaneous bioelectrical activity. Since hypoxia had the most pronounced effect on the viability and calcium activity parameters, this factor was chosen to assess the effect of Nec-1 application on the spontaneous bioelectrical activity of primary hippocampal cultures. The use of multi-electrode arrays for recording extracellular action potentials allowed to assess the functional integrity of neural networks in the posthypoxic period. It was shown that hypoxic injury leads to irreversible inhibition of the spontaneous bioelectrical activity of the primary hippocampal cultures’ neural networks by day 7 of the posthypoxic period (Figure 4). On day 7 of the posthypoxic period (21 DIV), the number of network bursts of impulses significantly decreased (“Sham”—36.12 ± 4.27 bursts/10 min; “Hypoxia”—15.87 ± 3.03 bursts/10 min, Figure 4A). The number of spikes in bursts also significantly decreased: from 90.22 ± 12.32 (Sham) to 11.58 ± 0.70 (Hypoxia), Figure 4B. It indicates the loss of a part of the functionally significant elements of the neural network.

RIP kinase inhibition had a pronounced protective effect on the bioelectrical activity of hippocampal cultures. On day 7 after modeling hypoxia, the number of spikes in the burst in the “Hypoxia + RIPK inhibitor” group (27.68 ± 5.50, Figure 4B) was significantly higher than in the “Hypoxia” group, and the number of small network bursts in the “Hypoxia + RIPK inhibitor” group (23.49 ± 2.14 Figure 4A) did not differ from the group of intact cultures.

**Figure 5:**
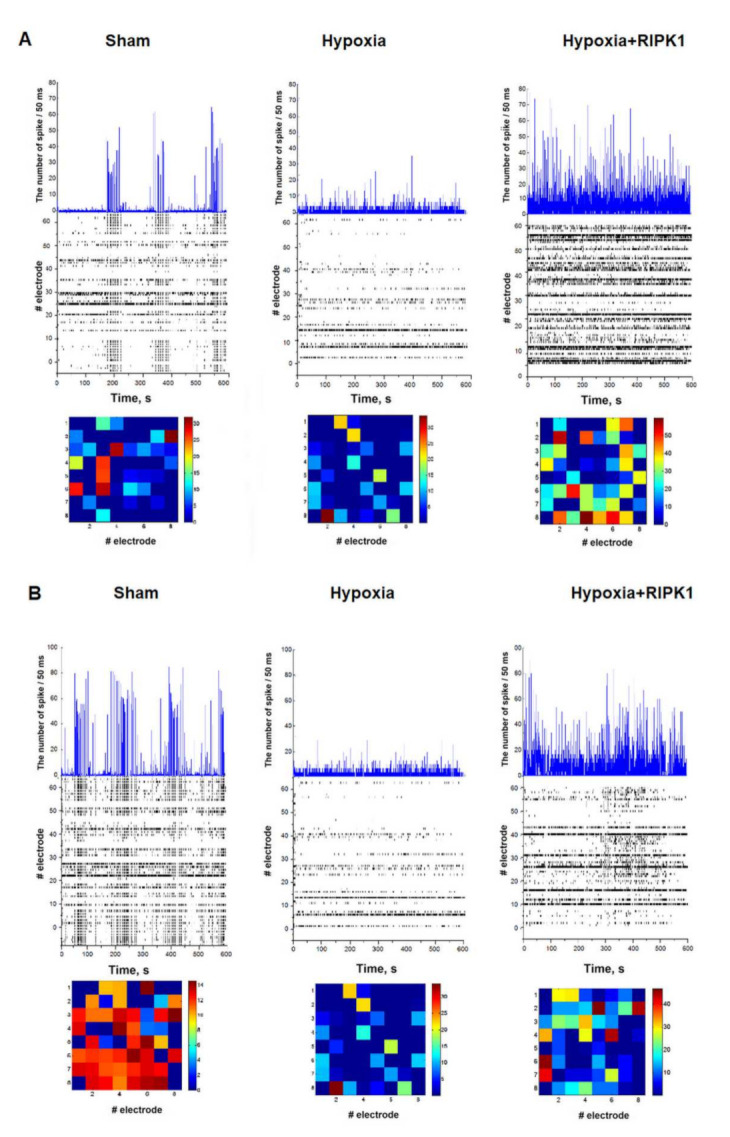
The number of spikes in 50 ms and typical examples of raster diagrams of spontaneous bioelectrical activity of primary neuronal cultures (top) and representative examples of patterns of spontaneous bioelectrical activity of primary neuronal cultures (bottom) on days 1 (**A**) and 7 (**B**) after modeling hypoxia. Color diagram—the time of spikes’ occurrence in the network burst, recorded from the electrodes, ms.

The analysis of activation patterns also revealed an increase in the time of impulse propagation between the electrodes after modeling hypoxia. At the same time, RIP kinase blockade retains the activation pattern and the burst structure on the first day after hypoxia (Figure 5).

In this way, the use of the RIP1 kinase inhibitor necrostatin-1 contributes to the partial preservation of spontaneous bioelectrical activity of the neural network in hypoxic damage.

### 2.2. Effect of RIPK1 Blockade on Resistance of Laboratory Animals to Hypoxic and Ischemic Brain Damage

The next stage of the study was to confirm the neuroprotective effect of the RIPK kinase blocker shown in vitro in the experimental modeling of hypoxia in vivo. The survival rate and the main parameters characterizing the animals’ resistance to hypoxic damage are presented in Table 1. The administration of necrostatin by intraventricularly injection demonstrated a pronounced protective effect.

It was shown that the survival rate of animals with intraventricular administration of necrostin-1 increased from 21.1% (AHH) and 25% (PBS) to 55.6% (RIPK1). Intraventricular administration of Nec-1 by control resulted in a significant increase in the survival time at altitude (AHH—3.8 ± 0.71 min.; RIPK1 inhibitor—7.65 ± 0.41 min.).

As for the degree of resistance to hypoxia, in both AHH and PBS groups, medium-resistant animals prevailed. The share of highly resistant animals in the AHH group was 21% and, in the PBS group, 8.3%. Intraventricular administration of the RIPK1 inhibitor increased the proportion of highly resistant animals to 33.3% (medium resistant—66%), and there were no low-resistant ones.

Assessment of the general motor and orientation-exploratory activity 24 h after the AHH modeling revealed no significant changes in the activity level of the animals (Table 2).

In animals that received an intraventricular injection of Nec-1 before AHH modeling, long-term memory was assessed using the Morris water-maze test 7 days after the hypoxic episode. It was revealed that in the distant posthypoxic period (7 days after AHH) in the “Intact” group, there was no significant decrease in mnestic functions, and the delayed coefficient of memory retention did not change (Table 3). There were no significant changes in the state of long-term memory of the animals in the posthypoxic period. It is interesting to note that the total distance that the animal swam in the maze in 1 min significantly decreased after AHH modeling, which may indicate a decrease in the general physical tone of the animals.

Therefore, to assess the neuroprotective effect of RIPK1 blockade in more detail, we modeled a more severe ischemic brain injury by performing unilateral irreversible carotid artery occlusion in mice. A pronounced neuroprotective effect of RIP1 kinase blockade was shown. The survival rate of animals in the “Ischemia” group was 66%, with intraventricular administration of the RIPK inhibitor necrostatin—83%.

We analyzed the motor and orientation-exploratory activity of the animals 24 h after modeling ischemic damage. The animals in the “Ischemia” group showed a significant decrease in the level of orientation-exploratory activity (the number of upright postures: “Intact”—40.0 [30.0; 40.0] and “Ischemia”—20.0 [5.0; 22.0] (Table 2). The “Ischemia + RIPK1” group of animals did not differ from the “Intact” ones. Thus, the RIPK1 kinase inhibition increases the resistance of mice to ischemic injury.

Analysis of memory traces during the reproduction of navigation learning in the Morris water maze showed that ischemia from which mice suffered previously led to more pronounced memory impairments than hypoxia.

In the “Intact” group, no failed attempts were detected on the third, fourth, and fifth sessions. In the “AHH” group, one animal failed to find the hidden platform on the third learning session. One animal was noted to have one failed attempt of three on the fifth learning session. In the “Ischemia” group, two animals failed one attempt to find the hidden platform on the third learning session and one attempt to find the hidden platform on the fifth learning session. In both groups with intraventricular Nec-1 injection (AHH + RIPK1 inhibitor and Ischemia + RIPK1 inhibitor), no failed attempts were detected on the fifth session. In the “Ischemia+ RIPK1 inhibitor” group, one animal failed one attempt to find the hidden platform on the third learning session.

The delayed coefficient of retention in the control group was significantly reduced compared to intact animals (“Intact”—41.62 ± 2.29; “Control”—29.2 ± 3.94). In the RIPK1 kinase blockade, the delayed coefficient of retention values did not differ from the parameters of intact animals (Table 3).

To sum up, in vivo studies confirmed that RIPK1 kinase blockade by the necrostatin-1 blocker increases the body’s adaptive capabilities in ischemic brain damage.

### 2.3. Histopathological Evaluation

**Figure 6:**
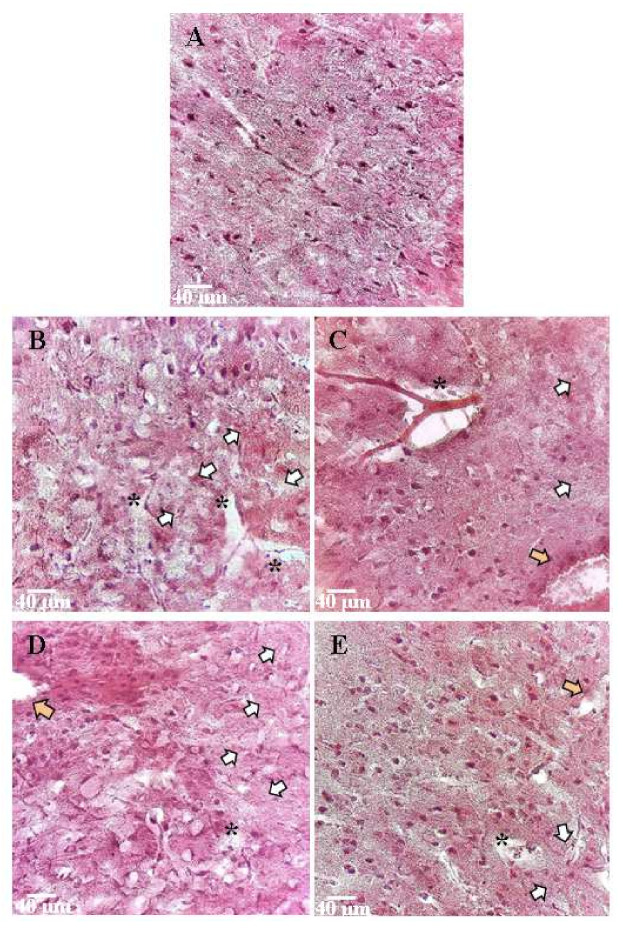
Representative microphotographs of histological specimens of the cerebral cortex of mice in the modeling of acute hypobaric hypoxia and ischemia. Hematoxylin-eosin staining, magnification ×40. (**A**)—Intact, (**B**)—Hypoxia, (**C**)—Hypoxia + RIPK inhibitor Necrostatin-1, (**D**)—Ischemia, and (**E**)—Ischemia + RIPK inhibitor Necrostatin-1. *—indicate the expansion and edema of the pericapillary space. White arrows indicate edema of the nerve tissue and nerve cells with indistinct edges or unformed nerve cells. Orange arrows indicate areas of hemorrhage and inflammation with pronounced cellular infiltration. Scale bar 40 µm.

Hematoxylin and eosin staining of brain tissue was performed in order to study morphological changes in the nervous tissue when using Nec-1 in modeling hypoxic and ischemic damage. The sensorimotor cortex and the area of the cerebral ventricles, where the most pronounced changes were observed, were selected for the study. In the “Intact” group, the cells were predominantly regular, round, or oval, with a diameter of 5.2 ± 0.6 µm. The nuclei had an elongated oval shape. The cytoplasm of cells was homogeneous, the contours of the cells were even, and the outgrowths were quite numerous (Figure 6A).

When AHH was modeled, microphotographs showed structural changes in the brain substance (Figure 6B). There was an expansion of the pericapillary spaces and hyperchromia of neurons, as well as an accumulation of unformed nerve cells near the expanding capillaries. The tissue swelling was pronounced. A significant part of the cells of the granular layer of the cerebral cortex had irregular shapes with uneven contours, and nuclear pycnosis was noted. The cell size was increased compared to the “Intact” group and was 8 ± 1.9 μm.

In case of RIP1 blockade, morphological changes were less pronounced than in the “Hypoxia” group. There were single altered neurons near the inflammatory focus. At the same time, there were cells with distinct contours of the nucleus and soma (15 ± 3%). Perivasal edema was less pronounced. The cortical cell size in the group with RIP1 kinase inhibitor was 7.5 ± 2.1 μm, which did not differ from the indices of the “Intact” group. There were light-colored neurons, which is normal for nerve tissue.

When ischemic damage was modeled in the “Ischemia” group, significant changes in the brain-tissue morphology were observed. Cytotoxic edema was pronounced strongly. In the area of the ventricles, there were areas of tissue separation. There was a plethora and thinning of the walls of blood vessels. Neurons of a light phenotype predominated due to swelling, and the cell diameter was increased to 9.1 ± 0.9 μm. In the brain ventricles’ area, there were areas of nervous tissue separation (Figure 6D).

As in hypoxia, the RIP1 kinase inhibitor necrostatin-1 had a positive effect on tissue structure when modeling ischemia. A decrease in edema in the cerebral cortex was noted, although there still were areas of hemorrhage. The granular and pyramidal layers’ cells had a shape close to normal, with clear, even boundaries; they also had a high nuclear-cytoplasmic ratio. The average cell diameter was 8.3 ± 1.8 μm. Some areas of regeneration were detected (Figure 6E).

**Figure 7:**
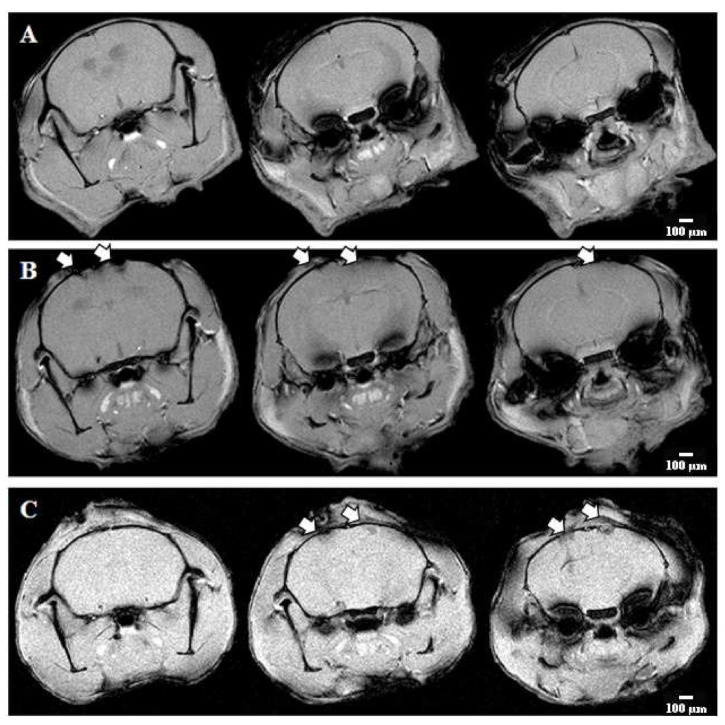
Representative MRI scans of the brain of mice in the modeling of acute ischemic brain injury. (**A**)—Intact, (**B**)—Ischemia, and (**C**)—Ischemia + RIPK inhibitor Necrostatin-1. White arrows indicate the active areas of ischemic lesion’s influence.

Magnetic resonance imaging (MRI) was performed on day 7 after ischemia modeling to assess the volume of ischemic focus and brain structures. The MRI scans of the intact brain showed that the cerebral hemispheres had a normal structure and intensity of the MR signal (differentiation of gray and white matter) (Figure 7).

When modeling ischemic damage, MRI scans of the control group demonstrated edema in the area of the sensorimotor cortex and ventricles of the left and right hemispheres. The volume of the affected area was 26.6 ± 7.4 mm^3^. The group with RIP1-kinase inhibitor necrostatin-1 showed less pronounced tissue structure disruption and a decrease in diffuse edema. However, the volume of the affected area did not differ from the “Ischemia” group and amounted to 26.2 ± 5.1 µm^3^.

To sum up, the sensorimotor cortex tissues of the cerebral hemispheres in the Nec-1 group had fewer pathological morphological changes.

## 3. Discussion

There is emerging evidence from recent studies indicating that the recently described type of cell death, necroptosis, plays an important role in the development of nerve cell damage associated with hypoxia, including in ischemia, traumatic brain injury, and neurodegenerative diseases.

Necroptosis is mediated by RIP1/RIP3/MLKL (receptor-interacting protein kinase 1/receptor-interacting protein kinase 3/mixed lineage kinase domain-like protein) signals. Under physiological conditions, RIPK1 kinase is usually inactive. The activation of RIPK1 kinase is caused by the stimulation of death receptors by external signals. These receptors include the tumor necrosis factor receptor (TNFR) 1, TNFR2, TLR3 and 4, as well as the Fas ligand [20]. Phosphorylation of RIPK1 at the Ser166 residue leads to the formation of its complex with death receptors (complex-I), followed by the activation and recruitment of RIPK3 and the formation of complex II. Then, downstream RIPK3-mediated phosphorylation of mixed lineage kinase like protein (MLKL) occurs, which leads to the formation of a necroposome.

Recently, it was shown for the first time that p-RIPK1- (Ser166) and RIPK3/MLKL-dependent necroptosis pathways are activated in the modeling of ischemic stroke [11]. These data allow us to take a new look at the molecular pathogenetic pathways of hypoxic ischemic damage to nerve cells and consider RIP1 kinase as a potential therapeutic target for neuroprotection. There is also evidence of the important role of necroptosis in neurodegenerative processes in Alzheimer’s and Parkinson’s diseases, as well as in the development of secondary injuries in traumatic brain injury [7,21].

However, necroptosis is a programmed type of necrosis that can be regulated and reversed under certain conditions. Necrostatin-1 is a selective inhibitor of RIPK1, but not RIPK3, which can prevent cell death by the necroptosis pathway. A recent in vitro study reported that Nec-1, but not iNec, specifically inhibits phosphorylated RIPK1 kinase at several residues, including Ser14, Ser15, Ser161, and Ser166 [22]. Thus, it can be used for selective pharmacological blockade of necroptotic cell death. Several recent publications have reported on the effectiveness of this approach in vivo in modeling ischemic stroke and neurodegenerative diseases.

For example, it has been demonstrated that RIPK1 knockout mice exhibit greater resistance to ischemic stroke [23]. Deng et al. demonstrated that the use of Nec-1 inhibited ischemia-induced increases in the RIPK3, MLKL, and p-MLKL expression, and it also reduced the volume of ischemic brain damage [18]. DTIO, the analogue of necrostatin, reduced infarct volume and improved neurological deficits in the acute phase after permanent middle cerebral artery occlusion (pMCAO), in addition to decreasing cerebral atrophy and promoting the restoration of brain function in the chronic phase of post-cerebral ischemia/reperfusion (I/R). In vitro application of DTIO reduced necroptotic cell death from oxygen-glucose deprivation (OGD) or oxygen-glucose deprivation and reoxygenation (OGD/R) caused by neuronal or astrocyte damage [24].

In RIPK1 or RIPK3 knockout mice, a reduction in the area of damage after traumatic brain injury up to 80% was demonstrated. At the same time, a decrease in astrocyte and microglia activation and an improvement in memory function were noted [12]. The authors suggest that progressive chronic brain damage and cognitive decline after TBI depend on RIPK1/3 expression in neurons. Probably, the neuroprotective effects of necrostatin-1 in ischemic brain damage are associated, among other things, with the inhibition of inflammatory reactions. Moreover, RIPK1 inhibition prevented mitochondrial fragmentation, a characteristic feature of necrotic cell death, in vitro and in vivo [25]. Interestingly, there is evidence that activation of RIP1 kinase can initiate two pathways of cell death: primarily RIPK3/MLKL-dependent necroptosis and Fas-associated protein with death domain (FADD)/caspase-8-dependent apoptosis. Dojo Soeandy et al. also demonstrated that inhibition of RIPK1 decreases the activation levels of both caspase-3 and caspase-7, indicating a relationship between the necrotic and apoptotic pathways in ischemic stroke [26].

Increased levels of RIPK1 expression and activation were demonstrated in cellular and animal models of Parkinson’s disease. RIPK1 inhibitor Nec-1s reduced the levels of apoptosis and cell necrosis, inflammatory responses, ROS production and mitochondrial dysfunction, and also reduced behavioral disorders in mice [27].

It is also hypothesized that the chronic inflammation observed in Alzheimer’s disease is associated, among other things, with increased production of cytokines of the tumor necrosis factor (TNF) family. TNF binds to TNFR1 and TNFR2 receptors to activate a variety of cellular responses that can be neuroprotective or neurodegenerative. In particular, TNF can induce the activation of RIPK1 and downstream cascades and trigger necroptosis, although its significance in the loss of neurons in AD is still largely unstudied [21]; however, it opens another potential therapeutic aspect of the use of necroptosis inhibitors.

Our study was the first to examine the effect of the RIP1 kinase inhibitor Nec-1 on the neural-network level. It is extremely important since even in those neurons that did not die as a result of hypoxic-ischemic damage, the processes of synaptic transmission can be disrupted, and synaptic contacts can degrade. It leads to disruption of the neural network activity, which underlies the cognitive impairments caused by ischemic damage.

Functional network activity is the emerging spatio-temporal patterns of transmission of spontaneously generated bioelectric and metabolic signals between nerve cells in functional neural ensembles. This rhythmically synchronized activity is essential for a variety of brain functions, including sensory or computational processing, decision making, consciousness, and memory [14]. Neurons transmit electrical impulses to each other, but the dynamic of calcium ions is also important for describing neuroglial networks. In our study, using multi-electrode arrays for long-term cultivation, we showed that the use of the RIP1 kinase inhibitor necrostatin-1 maintains the number of network bursts of impulses in hippocampal cell cultures in the posthypoxic period and also partially preserves the burst’s structure.

Calcium ions play a key role in the signal transmission between nerve cells, as well as in astrocyte–neuron and astrocyte–astrocyte signaling. Visualization of calcium dynamics using imaging methods allows to assess the functional state of neural networks with cellular resolution. In this manuscript, we used an algorithm of imaging data analysis developed by us and based on correlation analysis, which allows to reconstruct the dynamic architecture of neuron-glial networks [18,19]. It is an effective analytical tool for characterizing the collective and coordinated activity of neuron-glial networks at the functional level, as well as for assessing the impact of damaging and protective factors.

We were surprised to find that despite the preservation of neural network bioelectrical activity and the number of cells in which calcium events were recorded, the correlation of calcium dynamics with RIPK1 inhibition decreased significantly in the posthypoxic period. This is probably due to the fact that astrocytes begin to make a significant contribution to the calcium dynamics of hippocampal cultures after exposure to stress factors while their collective dynamics are being disturbed. Revealing the exact molecular and cellular mechanisms of this phenomenon is a field for further research.

In addition, to confirm the data obtained in vitro, we investigated the effect of Nec-1 on the resistance of small laboratory animals to the modeling of cerebral hypoxia and ischemia. It was revealed that intraventricular administration of Necrostatin-1 (RIPK1 kinase inhibitor) increased the resistance of C57BL mice to both hypoxic and ischemic brain damage and also reduced morphological damage to the nervous tissue. These data correspond well with the previously reviewed literature.

The results of our studies confirm that the inhibition of necroptosis can be the basis for enhancing the adaptive capabilities of nerve cells for the development of new strategies for correcting the consequences of exposure to damaging ischemic factors.

## 4. Materials and Methods

### 4.1. Research Object and Ethics Statement

Primary neuronal cultures obtained from the embryonic brain tissue of 57BL/6 mice (on the 18th day of gestation) were used for in vitro studies [25]. In vivo studies were carried out on 88 male C57BL/6 mice. All studies were performed on males to exclude the influence of the hormonal cycle phases on the experimental results. In order to exclude the influence of circadian rhythms, the experiments were carried out at the same time of day.

The rules for the care and use of experimental animals complied with the “Rules of working with laboratory animals” (Russia, 2010) and the “International guiding principles for biomedical research involving animals” (CIOMS and ICLAS, 2012). The ethical principles established by the European Convention for the Protection of Vertebrate Animals used for Experimental and Other Scientific Purposes were also respected (Strasbourg, 2006). The study was approved by the bioethics commission of the Lobachevsky State University of Nizhny Novgorod.

### 4.2. Isolation of Murine Primary Hippocampal Cultures

Obtaining and long-term cultivation of primary neuronal cultures were carried out in accordance with the protocol described by [25]. Surgically extracted embryonic brain tissue was subjected to mechanical and then enzymatic dissociation by 20-min incubation in 0.25% trypsin solution (Life Technologies, Carlsbad, CA, USA). The obtained cell suspension was applied to coverslips (18 mm × 18 mm) pretreated with a positively charged polyethyleneimine, hydrophilic substance (1 mg/mL, Sigma Aldrich, St. Louis, MO, USA), to ensure effective attachment of cells to the culture substrate. The initial cell density was 4500 cells/cm^2^. Primary neuronal cultures were cultured in NeurobasalTM medium (Invitrogen, Waltham, MA, USA), supplemented with 0.5 mM L-glutamine (Invitrogen, USA), 2% bioactive supplement B27 (Invitrogen, USA), and 5% fetal calf serum (PanEco, Moscow, Russia) in a CO_2_ incubator Binder (BINDER GmbH, Tuttlingen,Germany) at 35.5 °C and a gas mixture containing 5% CO_2_ for 21 days. The features of neuron-glial-network formation were initially assessed using an inverted fluorescence microscope Axio Observer A1 (Zeiss, Jena, Germany) [25].

### 4.3. Modeling of Ischemic Factors In Vitro

Modeling of glucose deprivation (GD) was carried out according to the previously developed protocol [14] by replacing the culture medium with a NeurobasalTM medium of equivalent composition, but containing no nutrient substrates (glucose, lactate, pyruvate), developed at the request of the project team by “PanEco” (Russia). After 60-min incubation, the medium was replaced with a conditioned medium. The “Intact” group of cultures underwent a complete replacement of the medium with a full-fledged growth medium. The RIPK1 inhibitor necrostatin-1 (Nec-1) (Sigma-Aldrich, Taufkirchen, Germany) at a concentration of 1 μm was applied to the experimental groups of cultures 20 min before modeling GD, during GD, and immediately after the reverse medium change. The control group of cultures was similarly supplemented with dimethyl sulfoxide (DMSO), a solvent, at a corresponding concentration.

Modeling of acute normobaric hypoxia was carried out by complete replacement of the culture medium with a low oxygen content (0.37 mL/L) [25]. Oxygen was displaced by saturating the culture medium with argon, an inert gas. The experiment was carried out in a sealed chamber in which air was also replaced by argon. The incubation time for primary hippocampal cultures was 10 min. The addition of Nec-1 was performed according to a scheme similar to the GD modeling. The number of independent biological repeats in each group was at least three.

### 4.4. Assessment of Cell-Culture Viability

To assess the viability of primary cultures, 7 days after modeling stress factors, cells were stained in vitro with specific fluorescent dyes: propidium iodide (Sigma Aldrich, St. Louis, MO, USA) and bisbenzimide (Sigma Aldrich, St. Louis, MO, USA), which allow visualizing the nuclei of dead cells and the total number of cells in the culture, respectively [17,28].

### 4.5. Calcium Imaging

The features of the functional metabolic activity of cells in primary neuronal cultures were studied using the Ca^2+^ imaging technique. This technique for recording fluctuations in the concentration of Ca^2+^ ions in the cytoplasm of nerve cells allows visualizing the functional architecture of neural networks at the cellular level and is one of the most informative methods for studying the metabolic activity of the entire neuron-glial network and its elements (neurons and glial cells) [29,30]. Oregon Green 488 BAPTA-1 AM (OGB1) dye (Termofisher, Waltham, MA, USA) was used as a calcium sensor. Visualization was performed using a confocal scanning microscope Zeiss LSM 800 (Zeiss, Jena, Germany) with a Plan Apochromat ×20 objective. Time series of OGB1 fluorescence field images were recorded. Calcium events (oscillations) were analyzed using the original software package “Astroscanner” (state registration certificate for computer software No. 2014662670). The records of the average OGB1 fluorescence intensity function of time F(t) were analyzed. To determine the start time (Tstart) and the end time (Tend) of the oscillation, the threshold of deviation from the mean value in the amount of the standard squared error F(t) was taken. The following parameters were estimated: duration (s) and frequency (number of calcium events/min) of calcium oscillations and the share of working cells in culture. At least three independent experiments were carried out, for each of which the registration of calcium activity was performed in at least three fields of view.

To study the features of the reorganization of the calcium dynamics of neuron-glial networks associated with the kinase blockade and the modeled ischemic factors, an original algorithm for the analysis of network characteristics of calcium activity in the AstroLab software was used [17,18], with a state registration certificate for computer software No. 2021612870 dated 25.02.2021.

The algorithm considers the entire image plane as a whole and identifies events as connected spatio-temporal areas with significant activity. Calcium events in individual cells of the network are analyzed by segmenting the image using the watershed algorithm. The initial events found are divided into individual cell regions. Further calculations are performed on the events found in each cell.

A simple dynamic neural-astrocytic network is represented as an undirected graph $G=\left(V,E\right)$, where the set of vertices V of which corresponds to the set of cells and the set of edges E to the correlations between the cells:

(1) $$V=\bar{1,\;n},\;\;\;\;E=\left\{\left(i,j\right)\;:i,j\in V,\;\;\;\;\rho_{ij}>\rho_{thr}\right\},$$

where $\rho_{ij}$—Pearson correlation coefficient between pairs of cells i, j∈V:

(2) $$\rho_{ij}=\frac{\sum_{k=1}^{n}{\overset{˘}{x}}_{k}^{i}\cdot {\overset{˘}{x}}_{k}^{j}}{\sqrt{\sum_{i=1}^{n}{\left({\overset{˘}{x}}_{k}^{i}\right)}^{2}\cdot \sum_{i=1}^{n}{\left({\overset{˘}{x}}_{k}^{j}\right)}^{2}}},$$

(3) $${\overset{˘}{x}}_{k}^{i}=x_{k}^{i}-\langle x_{s}^{i}\rangle {}_{k-w,k}$$

$x_{k}^{i}—$ characteristic of the i-cell at time k,

${\overset{˘}{x}}_{k}^{i}—$ characteristic minus the moving average with a w-window.

An individual cell is characterized by dynamic changes in intracellular calcium concentration $C_{k}^{i}$, as well as the dynamics of the size of the event in the cell $A_{k}^{i}—$ the number of active pixels. Based on these two characteristics, the correlation coefficient is calculated. For each pair of cells, the correlation function between the two cell characteristics is calculated, from which the moving average is subtracted.

This approach does not consider the possible delay in calcium events between cells. Taking this feature into account, we define a directed graph with the same set of vertices and edges, constructed on the maximum value of the correlation level at various possible signal shifts in the range of 10 frames (W), as a dynamic neural-astrocytic network. The directionality of the bond is defined as the direction of the time shift at the maximum point.

(4) $$V=\bar{1,\;n},\;\;E=\left\{\left(i,j\right)\;:i,j\in V,\;\;{\overset{˘}{\rho }}_{ij}>\rho_{thr},\;\;\tau_{ij}>1\right\}$$

(5) $$\rho_{ij}\left(\tau \right)=\frac{\sum_{k=\max\left(-\tau ,1\right)}^{n-\max\left(\tau ,0\right)}{\overset{˘}{x}}_{k}^{i}\cdot {\overset{˘}{x}}_{k+\tau }^{j}}{\sqrt{\sum_{i=1}^{n}{\left({\overset{˘}{x}}_{k}^{i}\right)}^{2}\cdot \sum_{i=1}^{n}{\left({\overset{˘}{x}}_{k}^{j}\right)}^{2}}},$$

(6) $${\overset{˘}{\rho }}_{ij}=\underset{-W\leq \tau \leq W}{\max}\rho_{ij}\left(\tau \right),$$

(7) $$\tau_{ij}=\underset{-W\leq \tau \leq W}{\mathrm{argmax}}\rho_{ij}\left(\tau \right).$$

where $\rho_{ij}\left(\tau \right)$—correlation coefficient between signals with a shift τ,

${\overset{˘}{\rho }}_{ij}$—maximum correlation coefficient at various shifts in the range $\left[-W;W\right]$,

$\tau_{ij}$—shift of signals at the maximum level of correlation.

Several characteristics of the resulting networks were considered for network analysis:

1. The number of functional connections between pairs of cells

(8) $$N_{c}=\left|E\right|;$$

2. Average number of connections per cell

(9) $$N_{n}={\left\langle \left|\left\{j\;:j\in V,\;\left(i,j\right)\in E\right\}\right|\right\rangle }_{i};$$

3. Average speed of signal propagation between cells

(10) $$S={\left\langle \frac{d_{ij}}{\tau_{ij}}\right\rangle }_{ij}$$

where $d_{ij}$–the distance between the centers of i- and j-cells; $\tau_{ij}$—signal delay;

4. Average network correlation

(11) $$Ρ=\langle \rho_{ij}\rangle {}_{ij};$$

5. Average level of neighboring cells’ correlation

(12) $${Ρ}_{a}=\langle \rho_{ij}\rangle {}_{ij},\;\left(i,j\right)\in E_{p},$$

where $E_{p}$—pairs of spatially adjacent cells;

We use the term “correlation” in this context in the following sense: the “correlation” between calcium fluctuations in cell pairs is calculated as the maximum of the cross-correlation function of signals over a fixed range of delays. This algorithm is part of the theory of digital-signal processing. Thus, we determined a measure of the similarity of signal behavior for pairs of cells. Since calcium-activity signals are not random values but the trajectories of some dynamic system, we do not expect a linear relationship between them. Without considering signal lag, cross-correlation values can be either negative or positive since the propagation of calcium between cells can create delays in the peaks of activity. However, selecting the optimal time lag allows us to identify the presence of the maximum level of cross-correlation between pairs of cells, which is a non-negative value.

### 4.6. Analysis of the Spontaneous Bioelectrical Activity of Primary Neuronal Cultures

To study the features of spontaneous bioelectrical activity of neural networks, the technique of non-invasive long-term registration of extracellular potentials using the multi-electrode system USB-MEA120-2-InVBC-System-E-Standard (Multichannel Systems, Germany) was applied. When a signal propagates through a neural network, multi-electrode systems allow to convert ion flows into flows carried by electrons (electric current), thereby registering even single electrical events—extracellular action potentials (spikes)—with maximum accuracy. Primary neuronal cultures were cultured on the multi-electrode array system MEA60 (Multichannel systems, Germany) according to the previously developed protocol [13]. The initial density of cells cultured on multi-electrode arrays was 9000 cells/cm^2^. Hypoxia was modeled on the 14th day of the cultivation according to the procedure above. The registration of spontaneous bioelectrical activity was carried out before the modeling of hypoxia, during hypoxia, immediately after the reverse replacement of the medium, and within seven consecutive days. The obtained electrophysiological data were analyzed in the MATLAB software environment using the original MEAMAN algorithm package (state registration certificate for computer software No. 2012611190). Particular attention was paid to network burst events. We analyzed small network bursts, the criterion of which was the occurrence of spikes on at least four different matrix electrodes with an interval not exceeding 100 ms [16]. The main parameters for evaluating burst events were: the number of network bursts in a record, the number of spikes in the burst, and the duration of the network burst. The features of the reorganization of the functional structure of the neural network response by constructing activation patterns determined by the time of occurrence of the spike sequence within a network burst of impulses were also investigated [31].

### 4.7. Administration of the Investigated Inhibitors In Vivo

Two methods of necrostatin-1 administration were tested in experiments in vivo. When injected intraperitoneally, Nec-1 was applied 40 min before modeling acute hypobaric hypoxia or ischemia at a dose of 25 μg/kg. The second protocol involved intraventricular administration at a dose of 10 μg/kg. The doses were determined based on previously published studies [17]. When intraventricular injection was performed, experimental animals were anesthetized with inhalation anesthesia (Isoflurane, Henry Schein, Great Britain). The surgical field was shaved and disinfected. A midline sagittal incision was made in the scalp with a scalpel. Then, two trepanation holes were drilled in the skull bones using a surgical drill, according to the coordinates corresponding to the localization of the left and right ventricles of the brain (AP −0.5 mm; ML 1.4/−1.4 mm) according to the Paxinos and Franklin’s mouse brain atlas (Paxinos, 2001). The stereotaxic injection was performed using a 5 μL Hamilton syringe with a 6S beveled needle into each cerebral hemisphere to a depth of 3.5 mm, while the injection rate of the drug was 0.2 μL/min and the volume was 1.5 μL. The mice were returned to their cages after complete restoration of motor activity.

### 4.8. Modeling of Acute Hypobaric Hypoxia In Vivo

Experimental animals were randomly divided into the following groups: “Intact” *n* = 19, “Control”—modeling of acute hypobaric hypoxia (AHH) with intraventricular administration of PBS *n* = 12, “AHH + RIPK1 inhibitor” *n* = 6, “AHH + SRC inhibitor” *n* = 6, and “AHH + IKKb inhibitor” *n* = 6. For AHH modeling, the animals were placed in a sealed pressure chamber, in which a pressure of 220–240 mm Hg was created, which corresponds to an altitude of 10,000 m above sea level. The “exaltation” was carried out at a speed of 183 m/s for 1 min. Hypoxia was modeled for each animal until the second agonal inspiration but not longer than for 10 min. The time from the moment of ascending to the required height until the agonal inspiration, the animal’s respiratory arrest, or after 10 min of being at the “altitude”, was estimated as the survival time. The following parameters were estimated: the time of animal’s posture loss, the time of survival at altitude, the time of posture recovery, as well as the survival rate and the degree of animal’s resistance to hypoxia. Animals with a survival time of less than 3 min were referred to the low-resistant group, from 3 to 7 min to the medium-resistant group, and more than 7 min to the highly resistant group.

### 4.9. Modeling of Ischemic Brain Injury In Vivo

Experimental animals were randomly divided into the following groups: “Intact” *n* = 6, “Control 2”—modeling of ischemia with intraventricular administration of PBS *n* = 12, “ischemia + RIPK1 inhibitor” *n* = 6, “ischemia + SRC inhibitor” *n* = 6, and “ischemia + IKKb inhibitor” *n* = 6. To model ischemic brain injury, we performed unilateral occlusion of the left carotid artery. The animals were anesthetized with isoflurane inhalation anesthesia. The hair was removed from the front surface of the neck, followed by an incision in the soft tissues. The left common carotid artery was isolated in the surgical field, after which the vessel was ligated with a non-absorbable ligature suture. Afterwards, the wound was sutured and sprinkled with streptocide bacteriostatic powder. After all the surgical procedures were carried out (it took 10–15 min), each animal was placed in a separate cage with free access to food and water.

### 4.10. The Open Field Test

The open field test was used to assess the animals’ locomotor activity, orientation-exploratory behavior, and anxiety. The test was performed one day after modeling AHH or ischemia in the IR Actimeter (PanLab, Spain). The infrared activity monitor IR Actimeter includes a two-dimensional 45 cm × 45 cm square frame and an infrared beam system for detecting the animal’s movements. The IR Actimeter allows testing the voluntary movement activity, the number and duration of standing up on the hind legs, stereotyped movements, and exploratory behavior in daylight and nighttime conditions. All parameters were recorded and analyzed using ActiTrack software (PanLab, Barcelona, Spain). The distance and time passed by the animals in the center and at the periphery of the installed arena were assessed, and the vertical motor activity of the animals as well as the number of grooming and defecation acts were recorded.

### 4.11. Morris Water-Maze Test

The Morris water-maze test was carried out in a circular pool (d = 90 cm) filled with opaque warm water. A movable platform (d = 10 cm) was placed in a certain place in the pool 1–2 cm below the water surface. The temperature of the water was 24–26 °C. The training was carried out after 5 days of the post-traumatic period. The animal-testing protocol consisted of five training sessions of three attempts to find a platform for each animal. The training was performed daily at the same time. During the experiment, the animal was placed in the maze in three different places relative to the platform, the location of which did not change during the entire testing period. The duration of each attempt was 60 s. If the platform was found, the animal remained on it for 15 s. If not detected, the animal was placed on the platform for the same amount of time. After being on the platform for 15 s, the animal was placed outside the labyrinth for the same time. Next, the cognitive functions of the animal were assessed 48 h after training was completed. Next, the cognitive functions of the animal were assessed. To do this, the platform was removed from the pool, and the duration of the animal’s stay in the area where the platform had been previously located was assessed for 1 min.

### 4.12. Histological Examinations

The material for the study was the areas of the rat cerebral cortex identified according to the cytoarchitectonic maps. For histological studies, the brain was fixed in a 10% formalin solution for 24 h at room temperature. The samples were then placed in 15% (for 24 h) and then in a 30% sucrose solution (for 24–48 h) at room temperature. Brain sections with a thickness of 10 μm were prepared using a freezing sliding cryostat Leica CM1520 (Leica Biosystems, Wetzlar, Germany). The prepared sections were stained with hematoxylin-eosin according to the standard technique (PanReac AppliChem, Darmstadt, Germany). Then, the sections were dehydrated in alcohols of ascending concentration, purified in xylenes, and embedded in a mounting medium (Consul-Mount, USA). The samples were examined using a Zeiss Primo Star light microscope (Zeiss, Jena, Germany) equipped with an Axio CamMRc camera (Zeiss, Germany). The preparations were photographed using a built-in digital camera at a magnification of ×10, ×20, and ×40. Cells were counted in ten fields of view in identical parts of the cerebral cortex of mice in different groups. The obtained images were analyzed using Fiji is just ImageJ program.

### 4.13. Magnetic Resonance Imaging

Magnetic resonance imaging was performed on a high-field magnetic resonance tomograph Agilent Technologies DD2-400 9.4 T (400 MHz) (England) with an M2M (H1) volume coil. The animals were warmed up with warm air with a temperature of 27 °C. The animals were under general anesthesia reached by Zoletil (Virbac, Carros, France) and Xila (“Interchemie werken “De Adelaar” B.V”, Netherlands) in the T1 and T2 weighted-image (WI) modes. Each animal was placed vertically using a holder to fix it inside the tomograph. A radiofrequency surface array coil was used to study the brain. VnmrJ software was used to obtain and process the results. The obtained tomographic images were analyzed using Fiji is just ImageJ program.

Layered sections of the brain in the frontal plane with obtaining T1 and T2 tomograms, weighted by proton density, were carried out applying a pulse sequence MGEMS (multi-gradient echo multi slice) with the following parameters: TR = 1000 ms; TE = 1.49 ms; number of echoes—six; FOV—20 × 20 mm; matrix—initially 128 × 128, and later 256 × 256; slice thickness—1 mm; number of slices—15; and scanning time—17 min 04 s. Next, a study was performed to obtain diffusion-weighted images of the animal brain. For this purpose, a pulse sequence SEMS + diffusion (spin echo multi slice) with the following parameters was used: TR = 1200 ms; TE = 2 ms; FOV—20 × 20 mm; the matrix—initially 128 × 128, and then 256 × 256; the slice thickness—1 mm; number of slices—15; scanning time—18 min.

### 4.14. Statistical Analysis

Quantitative results are presented as a mean ± standard mean error (SEM) for normal distributions or as a median value and second and third interquartile range. To compare the two unrelated groups, the Mann–Whitney U test and ANOVA were used. The Tukey post hoc test was used as a post hoc test following ANOVA. At least three independent biological replicates were used for all experiments. The difference between the groups was considered significant if the *p*-value was less than 0.05.

## 5. Conclusions

The effect of the RIPK1 blockade on the viability of neural-network activity of primary nerve-cell cultures in normal conditions and during modeling of ischemic factors was studied. It was shown that the use of the inhibitor Necrostatin-1 preserved the viability of cells of primary hippocampal cultures when modeling glucose deprivation in vitro. The bioelectrical and metabolic calcium activity of neuron-glial networks was assessed in vitro using calcium imaging and multi-electrode arrays.

It was shown that the RIPK1 blockade partially preserved the number of cells exhibiting calcium activity, as well as the bioelectrical activity of neuron-glial networks under the ischemic factors. It was demonstrated as well that the use of the RIPK1 kinase inhibitor Necrostatin-1 in vivo increased the resistance of C57BL mice to both hypoxic and ischemic brain damage.

## Figures and Tables

**Table 1:** The main indicators of animal resistance to acute hypobaric hypoxia (AHH).

Group/Test Score	Survival Time at Altitude, min	Survival Rate, %	Loss of Posture Time, s	Posture Recovery Time, s	Resistance, %
AHH (*n* = 19)	5.43 ± 0.35	21.1%	86.11 ± 1.89	679.67 ± 13.46	HR—21 MR—74 LR—5
PBS intraventricularly (*n* = 12)	3.8 ± 0.71	25%	76.83 ± 2.56	674.8 ± 53.13	HR—8.3 MR—58.3 LR—33.3
RIPK1 inhibitor intraventricularly (*n* = 9)	7.65 ± 0.41 *#	55.6%	79.4 ± 3.38	796.2 ± 64.36	HR—33.3 MR—66.0 LR—0

HR—high resistance, MR—medium resistance, and LR—low resistance. *—the differences are significant compared to the “AHH” group; #—the differences are significant compared to the “Ischemia” group; *p* ≤ 0.05, one-way ANOVA was followed by Bonferroni post-hoc test for multiple comparisons.

**Table 2:** The main types of motor and orientation-exploratory activity of animals after modeling acute hypobaric hypoxia and ischemia.

Group	Total Distance (mm)	Distance in the Central Zone (mm)	Distance in Peripheral Zone (mm)	Time Spent in the Central Zone (s)	Time Spent in the Peripheral Zone (s)	Number of Upright Postures
Intact (*n* = 6)	1606.1 [1456.7; 1611.2]	73.1 [63.7; 163.8]	1442.2 [1414.1; 1547.7]	16.4 [9.2; 16.4]	283.6 [283.6; 290.8]	40 [30; 40]
AHH (*n* = 5)	1636.7 [1399.1; 2167.4]	247.5 [108.2; 285.7]	1389.2 [1290.9; 1881.7]	24.0 [17.6; 39.6]	276 [260.4; 282.4]	38 [16; 43]
AHH + PBS intraventricularly (*n* = 5)	1515.6 [973.25; 2002.5]	359.1 [160.15; 480.65]	1355.45 [591.4; 1743.35]	49.5 [31.6; 152.1]	251 [147.9; 268.4]	22 [9; 27]
AHH + RIPK1 inhibitor intraventricularly (*n* = 6)	1339.4 [1314.3; 1633.4]	134.8 [128.7; 292.9]	1269.8 [1179.4; 1504.7]	24.92 ± 8.782 16.8 [11.6; 35]	283.2 [265; 288.4]	25 [11; 26]
Ischemia (*n* = 11)	1468.3 [296.6; 1558.1]	250.2[166.8; 250.2]	1302.4 [161.2; 1375.2]	24.4 [18; 38]	275.6 [262; 282]	20 [5; 22] * F = 3.37; *p* = 0.02
Ischemia + PBS intraventricularly (*n* = 6)	1468.3 [296.6; 1558.1]	262.1[147.9; 281.2]	1348.1 [482.7; 1444.2]	31.41 [24; 33]	243.3 [202; 271]	21 [4.5; 25.1] * F = 3.37; *p* = 0.044
Ischemia+ RIPK1 inhibitor intraventricularly (*n* = 6)	930.4 [756.2; 1555.5]	127. ± 32.73 109.3 [74.4; 166]	875.5 [674.6; 1445.7]	43.2 ± 28.09 16.8 [14.8; 21.6]	283.2 [278.4; 285.2]	17.6 ± 7.440 10 [9;25]

*—the differences are significant compared to the “Intact” group; one-way ANOVA followed by Bonferroni post-hoc test for multiple comparisons.

**Table 3:** The main indicators of delayed testing of experimental animals in the Morris water maze in modeling acute hypobaric hypoxia and cerebral ischemia.

Group/Test Score	Distance Covered before Platform Was Detected (cm)	Total Distance Covered (cm)	Delayed Coefficient of Retention (%)
Intact (*n* = 6)	13.21 [9.85; 15.2]	147.5 [135.94; 149.8]	42.68 [38.36; 43.14]
AHH (*n* = 5)	24.2 [13.45; 32.1]	117.48 [115.6; 124.44] * F = 7.256; *p* = 0.005	35.25 [32;38.36]
AHH + RIPK1 inhibitor (*n* = 6)	23.4 [12.9; 30.4]	141.47 [141.36; 150.09]	42 [41.87; 48]
Ischemia (*n* = 12)	28.5 [20.1; 34,1]	129.8 [125.64; 136.7]	29.14 [24.59; 33.09] * F = 4.66; *p* = 0.039
Ischemia + RIPK1 inhibitor (*n* = 6)	26.8 [16.7; 33.5]	147.28 [145.46; 151.15] # F = 7.256; *p* = 0.047	32.81 [24.53; 39.82]

*—the differences are significant compared to the “Intact” group; #—the differences are significant compared to the “Ischemia” group; one-way ANOVA was followed by Bonferroni post-hoc test for multiple comparisons.

## Data Availability

The data used to support the findings of this study are available from the corresponding author upon request.
